# Technology for sustainable living: The impact of digital inclusion on the health of China's elderly living alone

**DOI:** 10.1016/j.ssmph.2025.101751

**Published:** 2025-01-09

**Authors:** Yong Yan, Huixia Xing

**Affiliations:** a School of Insurance and Economics, University of International Business and Economics, 100029, Beijing, China; b School of Finance, Henan University of Economics and Law, 450046, Zhengzhou, China

**Keywords:** Digital inclusion, Older adults living alone, Mental health, Physical health, China

## Abstract

Enhancing elderly health has become an important measure for coping with population ageing and building a healthy China. Among them, older adults living alone seem to suffer from greater loneliness and psychological stress. We analyzed data from the China Health and Retirement Longitudinal Study in 2015, 2018, and 2020, and carried out an empirical investigation into the impact of digital inclusion on the health of elderly individuals living alone, using two-way fixed effects models and two-stage least squares. The findings suggest that digital inclusion could positively impact the health of older people living alone. Specifically, for a one-standard-deviation increase in digital inclusion, the depression scale score decreases by 0.48 (α = −0.21, p < 0.01); the cognitive function score increases by 0.27(α = 0.12, p < 0.01); the instrumental activities of daily living score would decrease by 0.11 (α = −0.05, p < 0.01). An increase of 1 in the digital inclusion score decreases the self-rated health score by 0.02 (α = −0.02, p < 0.01). It can significantly enhance the health status of elderly people who live alone through mechanisms of improving life satisfaction, increasing the utilization of preventive health care services, and promoting social participation. Subsequent analyses identified varying effects of digital inclusion on older adults living alone, influenced by their income and education levels. In particular, digital inclusion substantially enhanced instrument activities of daily living among the aged who lived alone and with higher income and educational backgrounds. However it had no significant effect on older people living alone with lower incomes and educational backgrounds. The insights from this study could be invaluable for policymakers in promoting broader adoption of digital technologies among older adults living alone.

## Highlights

• Using data from the China Health and Retirement Longitudinal Study, the study explores how digital inclusion affects the mental and physical health of a specific group of older adults living alone in China.
• Reveals that digital inclusion enhances the health of older adults living alone by improving life satisfaction, increasing preventive healthcare use, and promoting social participation.
• Finds a significant positive impact of digital inclusion on the instrumental activities of daily living for high income older adults living alone, but no significant effect on those with lower income or education levels.
• Provides actionable insights for policymakers to promote digital technology adoption among older adults living alone.
• Offers guidance for policy development not only in China but also in other rapidly aging and developing countries.

## 1 Introduction

As global aging accelerates, governments and societies worldwide are showing greater concern for the health of elderly populations (Prince et al., 2015). It reported that, by 2050, individuals aged 60 and above will account for 21.3% of the world's population. Data indicate that by 2050, those over 60 will make up 21.3% of the global population (United Nations, 2019). China's population is aging faster than that of industrialized nations like the US (Kinsella & Phillips, 2005, p. 15), and ‘its people tend to get older before they get wealthy’ (Gan et al., 2014, p. 19). The role of family care for the elderly is rapidly diminishing(Feng et al., 2020), even as the growth of social services for elder care is comparatively lagging (Wong & Leung, 2012). There has been a notable increase in the population of elderly individuals living independently. The number of elderly individuals living alone has risen sharply (Zeng et al., 2015), directly affecting their quality of life and the amount of stress they experience. According to 2020 data, the number of elderly individuals living alone in China has reached 37.29 million households, reflecting an increase of about 6.5% from 2010 (National Bureau of Statistics of China, 2021). Elderly people living alone are those who live alone due to widowhood, divorce or other reasons, without a partner or children (Lim & Kua, 2011). Research has demonstrated that elderly individuals who live alone are more susceptible to experiencing psychological challenges, including loneliness, depression, and anxiety (Joutsenniemi et al., 2006; Sok & Yun, 2011; Perissinotto & Covinsky, 2014). In addition, their physical health may deteriorate due to the lack of appropriate care and health information (Lindström, 2009; Cacioppo & Hawkley, 2009; Tabue Teguo et al., 2016; Zebhauser et al., 2015).

Numerous studies have confirmed that the health of older adults is closely tied to their engagement with internet. For instance, Meischke et al. (2005) employed descriptive statistical analysis and chi-square tests to examine whether older adults use internet to access information related to heart attacks, and found that older adults who accessed the internet were more knowledgeable about the symptoms and preventive steps of heart disease, which led to improved health behaviors. Cresci et al. (2012) investigated how internet use affects the health of urban seniors by analyzing data from a micro-survey centered on their internet habits. They found that urban elderly interviewed improved their health by browsing through health portals for advice and, thus, by conducting effective health management. Based on data from older adults in Alabama, Cotton et al. (2013) found that internet use might reduce loneliness and improve social interaction among elderly people in both assisted and independent living situations. Lee and Jang (2022) investigated the link between internet usage shifts and self-rated health among older and middle-aged individuals during the COVID-19 outbreak, concluding that higher internet engagement boosted their health ratings. Hofer and Hargittai (2024) observed that the relationship between internet use and anxiety and depression in older adults differs, especially in cases of depression, where the strength of the connection varies. Most of these studies have concentrated on developed nations, with a primary focus on older demographic groups.

In this regard, older adults who live alone may experience differences compared to those who cohabit with others. Firstly, older persons living alone face higher risks of digital exclusion due to inadequate computer skills and access to internet because of lack of formal and informal social support. Secondly, the negative emotions and feelings, such as anxiety, helplessness, and loneliness caused by social isolation and small living space are common among older people living alone. Combining this with their fragile psychological state turns them into one of the most vulnerable groups within society. Unlike senior citizens in general, elderly people living alone will also generally have fewer social resources. Thirdly, most of the elderly living alone come from rural areas with less educational background. Consequently, their level of digital literacy is lower, and with it, their level of digital health literacy. Unlike their urban peers, rural elderly living alone often engage with internet for recreational purposes, which limits the health-promoting potential of digital technologies. Currently, although there is substantial evidence that internet use can help alleviate social isolation or loneliness among older adults, further research is needed, particularly for this specific group of older individuals who live alone in developing countries.

To the best of our knowledge, only a few papers have explored the relationship between internet and older adults living alone. (Cho et al., 2024; Silva et al., 2022; Srivastava et al., 2021). However, there are still some research gaps that remain. Firstly, Some factors, including cognitive functioning and the Instrumental Activities of Daily Living (IADL, used to evaluate an older person's independence in daily life, including their ability to perform activities such as cooking, shopping, managing finances, etc.) of older persons living alone, have not yet been adequately taken into account, which may importantly be associated with internet use. Meanwhile, related studies have not yet fully revealed the mechanisms underlying this association. For this reason, this paper will take advantage of the data from CHARLS with important variables such as cognitive function and IADL, in order to further research the influence of digital inclusion on the mental and physical health of seniors living alone. It also discusses in detail how digital inclusion can improve the mental and physical health of older people living alone through the channels of life satisfaction, health service utilization, and social engagement. Secondly, in most prior studies, the measuring framework of internet use was relatively homogenous, usually focusing only on internet use or not, ignoring multidimensional factors such as the type of tools and skill level of internet use. This simplified analysis limits the explanatory power and applicability of the research findings. This paper refines internet use measure and thus updates its health effects for a nationally representative sample of older persons living alone from a digital inclusion standpoint. Thirdly, existing studies have mainly focused on developed countries and lack empirical analysis of digital inclusion in developing country contexts. This research bias not only ignores the unique socioeconomic contexts of developing countries, but also constrains the global reach of digital technologies. Within the framework of China's Healthy Strategy, this study focuses on older people living alone in developing countries, assesses the far-reaching impact of digital inclusion on their health, and reveals the important role of digital technology in addressing the challenges of rapid aging. The findings provide valuable insights for policy development, not only in China but also for other countries with rapidly aging populations and developing nations.

This paper is organized as follows: the second section introduces the theoretical framework; the third section reviews the data sources, variables, and model specifications; the fourth section tests the robustness of the empirical findings and investigates the various pathways through which digital inclusion affects the health of older adults living alone; and the last section offers a discussion and conclusion.

## 2 Conceptual framework

The concept of digital inclusion involves the acquisition, skill development, and the use of internet (Livingstone & Helsper, 2007). Acquisition refers to the availability of internet and digital devices, skills involve the ability to use these technologies effectively, and use refers to the degree of actual participation in digital platforms and services. In the literature, the division of digital inclusion into “usage layer” and “skills layer” has been widely recognized. There is currently no consensus on the controversy surrounding the third level of digital inclusion (Friemel & Signer, 2010). However, Most empirical studies tend to focus only on a single level, such as whether the elderly use internet, while ignoring the multi-level issues of digital inclusion. In response to this and referencing pertinent literature (Dewan & Riggins, 2005), the empirical investigation in this paper focuses mainly on the first two dimensions.

The Conservation of Resources Theory plays a vital role in explaining how people cope with stress (Hobfoll et al., 2018). It centers on the thought that the intrinsic goal of human beings is to obtain, maintain, develop, and protect those resources that carry value for them. Stress ensues from threats to core resources, the loss of resources, or when one fails to acquire resources after significant investment. For older people living alone, digital inclusion can be regarded as an important tool of resources. First of all, digital technologies can expand their social support networks by being more connected with the outer world, which has been shown to reduce loneliness (Lissitsa & Chachashvili-Bolotin, 2016; Shah et al., 2021). Secondly, the emergence of online health services such as remote medical consultations and health management apps has enabled digital technologies to enhance the physical health through timely medical support and health information (Cresci et al., 2010; Rubbio et al., 2020). Moreover, resource conservation theory maintains that a resource gain helps people in coping with any future potential stressors. The use of digital technology not only enhances the cognitive functioning (Kamin & Lang, 2020; Cho et al., 2023), but also strengthens their sense of meaning and role value in life (Hunsaker & Hargittai, 2018; Köttl et al., 2021). The accumulation of these resources can ultimately help older adults living alone build greater psychological resilience and thus cope more comfortably with life's unexpected events. Given this, the following hypothesis is put forward:

Hypothesis 1 Digital inclusion can improve the mental and physical of older adults who live alone.

There are three channels through which digital inclusion can impact the health of older adults who live alone. The first channel is that digital inclusion fulfills the autonomy and relatedness of older adults living alone by arousing their intrinsic and extrinsic motivation, which enhances their life satisfaction and therefore influences the health of older persons living alone. Life satisfaction is defined as an individual's assessment of the general quality of life according to their own criteria (Diener et al., 2013), and is an important dimension in measuring the subjective perceived health of older adults (Moons et al., 2006). Self-Determination Theory argues that individuals are capable of independently setting and achieving their goals, with their behavior shaped by both intrinsic and extrinsic motivations (Deci & Ryan, 2012). Intrinsic motivation is the internal motivation that arises from an individual's enjoyment, interest, or sense of satisfaction in achieving a task; extrinsic motivation comes from external factors such as external rewards, punishments, or social recognition. It can be said that when intrinsic motivation and extrinsic motivation are balanced in concert with the psychological needs of individuals, life satisfaction may be achieved to a higher degree. Digital inclusion has the potential to stimulate and integrate both intrinsic and extrinsic motivations, improving life satisfaction among older individuals living alone. First, the learning and use of digital technologies can stimulate intrinsic motivation in older individuals living alone by creating feelings of fulfillment and the satisfaction of skill acquisition. Second, digital technology offers support because of extrinsic motivation. For instance, emotional support and social recognition from online interactions with friends may raise the sense of societal belonging among older adults living alone. Thus, the following hypothesis is proposed:

Hypothesis 2a Digital inclusion helps older adults living alone by enhancing their life satisfaction.

The second channel is that digital inclusion boosts the mental and physical health of older adults living alone by facilitating greater engagement with utilization of preventive health care services. Utilization of preventive health care services means taking proactive steps to use healthcare resources that help prevent disease, improve health, catch health issues early, or reduce the risk of worsening health conditions (Tian et al., 2010). It is important for the elderly to know their health status as early as possible and to take timely preventive measures against diseases. Health Human Capital Theory argued that health is an investment good, and individuals can promote health by purchasing healthcare services and consuming leisure time (Grossman, 2017). Especially in older age groups, digital technology is an important means of enhancing healthy human capital. Digital technologies break geographical and time constraints and help older people living alone to access healthcare resources more easily (Choi & DiNitto, 2013), thus increasing the frequency of healthcare utilization and enhancing health status. Given this, the following hypothesis is put forward:

Hypothesis 2b Digital inclusion helps older adults living alone by increasing the utilization of preventive health care services.

The third channel is that digital inclusion can effectively enhance the health of older individuals living alone by increasing their participation in society. Social participation is a behavior and process by which an individual actively participates in social activities, establishes social contacts and integrates into social groups, either formally or informally (Levasseur et al., 2010). Social Participation Theory focuses on how individuals may gain psychological and emotional support from social activities that promote health (Thoits, 2011). Active social participation not only enhances social interaction with increased frequency but also strengthens the individual's self-efficacy and social identity to maintain psychological and physical health. It has been proved that socially engaged elderly people are usually more active in self-management and adaptation to life difficulties and cope well with the health, economic, and social burdens (Herzog et al., 2002). For seniors living alone, the use of digital technology creates a new means for social participation. Through social participation, they have the opportunity to alter their self-view and grow closer to society, which may result in overall improvements in their mental and physical health. Therefore, the following hypothesis is suggested:

Hypothesis 2c Digital inclusion helps older adults living alone by influencing their social participation.

## 3 Data sources, measurement and model

### 3.1 Data

The CHARLS is a representative tracking survey of person aged 45 and above in mainland China, which aims to build a high-quality public micro-database that collects multidimensional information on socio-economic and health status, in order to meet the needs of scientific research on aging. The design of the survey was done by referring to a series of international aging research studies, including the American Health and Retirement Study (HRS). This survey, which started in 2011, is an important tool for multidisciplinary research in public health, sociology, and economics. The household questionnaire of the CHARLS provides a great deal of household and individual data, and it has the following three major strengths in researching how digital inclusion affects the health of older individuals living alone: first, the sample of this survey is highly representative, covering 28 provinces, municipalities, and autonomous regions, which ensures the generalizability of the findings; second, this database is designed for the middle-aged and older population, covering key data on income, consumption, and healthcare expenditures needed for this study; and finally, it provides detailed information on mental health indicators, including depression, cognitive decline, and physical dysfunction. In this analysis, we applied all three waves of the CHARLS data (2015, 2018, 2020). Since this study concentrates on the health of elderly individuals living alone, the sample is confined to those aged 60 and older, excluding individuals with an income below 50 yuan. After eliminating observational data with missing values for significant variables, the total sample size amounted to 4069.

### 3.2 Measurement

The dependent variable is the health status, both mental and physical, of elderly individuals residing alone. Mental health refers to an individual's achievement of a state of well-being on the cognitive, emotional and behavioral levels of the psyche (Galderisi et al., 2015). Physical health means that an individual maintains a normal state of physiological structure and function (Salovey et al., 2000). The Center for Epidemiologic Studies Depression Scale (CES-D) and the Mini-Mental State Examination (MMSE, mainly evaluates cognitive function, covering orientation, memory, attentiveness, calculation, linguistic abilities, and visuospatial understanding) and were employed to assess mental health (Perreira et al., 2005; Norris et al., 2016) of elderly individuals living alone. Specifically, the CES-D 10-item scale was used to evaluate depression levels in the CHARLS data. The scale includes the following questions: (1) I feel as though something that normally doesn't bother me is haunting me; (2) I find it hard to concentrate on what I'm doing; (3) I feel depressed; (4) I think that everything I do is an effort; (5) I am full of hope for the future (reverse coding); (6) I feel afraid; (7) I have trouble falling asleep; (8) I am very happy (reverse coding); (9) I feel lonely; and (10) I find it impossible to move on. Respondents were asked how frequently these emotions and behaviors occurred over the past week, with response options ranging from “rarely or none of the time” to “most or all of the time,” corresponding to scores of 0, 1, 2, and 3. The worse the mental health, the higher the score and the higher the degree of depression. The depression score range, as determined by the scale, is 0–30 points. Mentally sound older persons can generally retain greater levels of perception, memory, reasoning, and decision-making. The gathering and processing of information, including memory and intellectual integrity, is a part of these cognitive functions. Following (Christelis et al., 2010; Xiaoyan & Hong, 2018), each correct answer is calculated to obtain the MMSE score ranging from 1 to 21. The stronger the cognitive function, the higher the score. Participants in the CHARLS study were instructed to read 10 common words and then recollect them in any order twice: the first time around and the second time around 4 min later. The final score for the overall word recall was calculated by averaging the correct responses to the immediate and delayed word recall tasks. Explicit memory is a crucial component of fluid cognition as a measure of it. Tests of date cognition, math, and drawing skills were used to assess the mental state in more detail. Physical health status of elderly individuals living alone was measured using self-rated health (SRH) (Eriksson et al., 2001), a five-category ordinal variable that ranges from “very good” to “very poor” (Scores are assigned between 1 and 5, where greater values denote lower self-rated health), alongside instrumental activities of daily living (IADL) (Gobbens and van Assen, 2014), assessed with a widely recognized scale from 1 to 15, where higher scores denote poorer performance in instrumental activities.

As mentioned above, this study utilizes a framework that categorizes digital inclusion into two dimensions: digital access and digital skills. Digital access evaluates whether older adults living alone can connect to internet through various devices. The assessment includes two questions: Whether to access internet, and whether to use desktop computers, laptops, tablets or mobile phones and other devices to access internet. Answers to the first question were recorded as “yes” or “no”, with scores of 1 and 0, respectively. The second question allows multiple answers, with each device used to access internet getting a score of 1. The total score of digital access is obtained by adding the scores of these two questions. The higher the score, the better the digital access. Digital skills refer to the ability of older adults living alone to handle various digital tools, such as online communication, reading news, watching videos, playing games, managing finances, using mobile payments, engaging on WeChat, and sharing posts in social groups. Digital skills are assessed by scoring 1 point per activity, and the cumulative score indicates the level of proficiency. The overall digital inclusion score is calculated by summing the scores for digital access and digital skills, with a higher score representing a greater degree of digital inclusion for older individuals living alone.

Based on existing literature and the questionnaire structure (Hargittai et al., 2019; Rengui et al., 2022; Ding et al., 2023), four types of control variables are selected for this study. The first category encompasses individual factors, including age, gender (male = 1, female = 0), educational level and household registration (rural = 1, urban = 0). The second category includes family-related factors such as household per capita income, the frequency of weekly meetings with children (at least once a week = 1, others = 0), and the number of living children. The third category covers behavioral factors, including smoking status (quilt = 1, no = 0), drinking status (quilt = 1, no = 0), pension insurance participation (yes = 1, no = 0) and health insurance participation (yes = 1, no = 0). The fourth category addresses health and medical services factors, such as the presence of chronic diseases (yes = 1, no = 0), doctors and inpatient beds per 1000 population in the region. Descriptions of these variables are thoroughly outlined in Table 1.

**Table 1:** Variable explanation and descriptive statistics.

Variables	Variable explanation	Mean ± SD/N(%)
Dependent variable:		
CES-D	Continuous variable	10.26 ± 7.04
MMSE	Continuous variable	10.98 ± 3.68
SRH	Feel very good	398(9.77%)
	Feel good	527(12.94%)
	Average	1876(46.11%)
	Feel not good	932(22.92%)
	Feel very poor	336(8.26%)
IADL	Continuous variable	5.99 ± 2.31
Independent variable:		
Digital inclusion	Continuous variable	0.88 ± 2.28
Control variables:		
Age	Continuous variable	72.50 ± 8.37
Gender	Male	1590(39.07%)
	Female	2479(60.93%)
Educational level	Below elementary school	2473(60.77%)
	Elementary school	793(19.50%)
	Middle school	498(12.24%)
	High school and above	305(7.49%)
Household registration	Rural	2574(63.27%)
	Urban	1495(36.73%)
Log(Household per capita income)	Continuous variable	8.58 ± 1.50
Frequency of meetings with children	At least once a week	1982(48.71%)
	Others	2087(51.29%)
Number of living children	Continuous variable	3.17 ± 1.64
Smoking status	Quit	984(24.18%)
	Still	3085(75.82%)
Drinking status	Quit	1095(26.90%)
	Still	2974(73.10%)
Pension insurance participation	Yes	3577(87.91%)
	None	492(12.09%)
Medical insurance participation	Yes	3799(93.37%)
	None	270(6.63%)
Chronic disease	Yes	3525(86.64%)
	None	544(13.36%)
Number of residential care beds per 1000 persons	Continuous variable	4.80 ± 1.70
Number of doctors per 1000 persons	Continuous variable	2.50 ± 1.06
Mechanism variables:		
Life satisfaction	Not satisfied at all	174(4.27%)
	Not very satisfied	400(9.82%)
	Somewhat satisfied	1918(47.14%)
	Very satisfied	1355(33.31%)
	Extremely satisfied	222(5.46%)
Utilization of preventive health care services	Taken a medical examination in the past year	2145(52.72%)
	None	1924(47.28%)
Social participation	Yes	1958(48.11%)
	None	2111(51.89%)

In order to further research the influence of digital inclusion on the health of the elderly living alone, this paper sets life satisfaction, utilization of preventive health care services and social participation as mechanism variables. The definitions of these variables are as follows: (a) Life satisfaction: Based on the CHARLS database's original survey question “DC026: Are you generally satisfied with your life? The responses varied from “not satisfied at all” to “extremely satisfied,” and the life satisfaction scores ranged from 1 to 5. This matches the following five responses found in the database: “not satisfied at all,” “not very satisfied,” “somewhat satisfied,” “very satisfied,” and “extremely satisfied.” (b) Utilization of preventive health care services: Whether one has undergone routine physical examination within the past year measures the utilization of preventive health care services. If a specific participation time is provided, the value is 1; otherwise, it is 0. (c) Social participation: This is determined based on responses to the questionnaire regarding whether the older “visit, interact with friends,” “taking part in leisure activities like mahjong, chess, or cards, or visiting community recreational spaces,” “provide assistance to relatives, friends or neighbors who do not live with them,” “dance, exercise, practice Qigong, etc.,” “taking part in club activities,” and “participate in volunteering, or caring for patients or people with disabilities who do not live with them.” If the answer to any of these questions is “yes”, the person is considered to have participated in a social activity, and the variable value is 1 if the response to any of these questions is “yes,” and it is 0 otherwise. This indicates that the individual has engaged in social engagement. The descriptive statistics for all the variables mentioned are shown in Table 1.

### 3.3 Model

The sample studied in this paper is from three surveys conducted across 28 provinces, autonomous regions, and municipalities nationwide. In addition, data at different times and regions do not meet the assumption of homogeneity in terms of technological, environmental, and social factors; therefore, this paper uses a fixed-effects regression model. The LSDV (Least Squares Dummy Variables) is used as the basic estimation method to avoid such problems as endogeneity, heteroskedasticity, omitted variables, and so on for the data characteristics. To control survey year and region fixed effects, this paper chooses in order to avoid some influence of unobservable exogenous variables in time and region on the explanatory variables. The model for conducting the baseline regression is as follows:

(1) $${\text{Health}}_{\text{ict}}=\alpha_{0}+\alpha_{1}DI_{\text{ic}t}+\beta^{\prime }Ζ+\lambda_{t}+\delta_{i}+\varepsilon_{ict}$$

Where *i*, *c*, and *t* represent the individual, city, and year, respectively. *Health*_*ict*_ is a four-item indicator for the health status of older individuals living alone: CES-D, MMSE, SRH, IADL. *α*_*0*_ is the intercept term while *α*_*1*_ is the core estimation parameter and *DI*_*ict*_ stands for the digital inclusion of older adults living alone. Where ***Z*** denotes the control variable for controlling four types of factors: individual, family, behavioral, health and medical services factors. *λ*_*t*_ and *δ*_*i*_ are fixed effects for city and year respectively, while *ε*_*ict*_ is a stochastic disturbance term. Meanwhile, there are three important mechanisms that will be discussed in this paper, namely, how digital inclusion can influence the health of older adults living alone through life satisfaction, utilization of preventive health care services, and social participation.

## 4 Results of empirical study

### 4.1 Results

Table 2 reports the impact of digital inclusion on the health of older people living alone. Columns (1) and (2) reflect the effects of digital inclusion on the mental health of older adults living alone, and columns (3) and (4) reflect the effects of digital inclusion on the physical health of older adults living alone, respectively. For a one-standard-deviation increase in digital inclusion, the CES-D score decreases by 0.48 (α = −0.21, p < 0.01, α∗SD = 0.21∗2.28 = 0.48), which is an increase equal to 4.7% (0.48/10.26∗100% = 4.7%) of the average depression score; the MMSE score increases by 0.27 (α = 0.12, p < 0.01, α∗SD = 0.12∗2.28 = 0.27), which is equivalent to 2.5% (0.27/10.98 ∗100% = 2.5%) of the average MMSE score; the IADL score would decrease by 0.11 (α = −0.05, p < 0.01, α ∗SD = 0.05∗2.28 = 0.11), this reduction corresponds to 1.8% (0.11/5.99 ∗100% = 1.8%) of the average IADL score. An increase of 1 in the digital inclusion score decreases the SRH score by 0.02 (α = −0.02, p < 0.01). This indicates that digital inclusion can significantly enhance depression and alleviate cognitive deterioration among older individuals living alone, and is also able to improve and enhance physical health. These findings support Hypothesis 1.

**Table 2:** Effects of digital inclusion on the health of older individuals living alone.

Variables	(1)	(2)	(3)	(4)
	Mental health		Physical health	
	CES-D	MMSE	SRH	IADL
Digital inclusion	−0.21∗∗∗	0.12∗∗∗	−0.02∗∗∗	−0.05∗∗∗
	(0.02)	(0.01)	(0.00)	(0.01)
Age	0.01	−0.06∗∗∗	0.00∗	0.06∗∗∗
	(0.01)	(0.01)	(0.00)	(0.00)
Gender	−1.74∗∗∗	0.48∗∗∗	−0.01	0.11∗∗∗
	(0.13)	(0.07)	(0.02)	(0.04)
Elementary school	−0.74∗∗∗	1.95∗∗∗	0.03	−0.21∗∗∗
	(0.14)	(0.08)	(0.02)	(0.04)
Middle school	−1.23∗∗∗	2.53∗∗∗	−0.05∗∗	−0.20∗∗∗
	(0.16)	(0.09)	(0.03)	(0.04)
High school and above	−1.60∗∗∗	3.01∗∗∗	−0.04	−0.15∗∗∗
	(0.20)	(0.10)	(0.03)	(0.05)
Household registration	1.30∗∗∗	−0.70∗∗∗	0.10∗∗∗	0.10∗∗
	(0.15)	(0.08)	(0.02)	(0.04)
Log(Household per capita income)	0.00∗∗∗	0.00	0.00	0.00∗∗∗
	(0.00)	(0.00)	(0.00)	(0.00)
Frequency of meetings with children	−0.45∗∗∗	0.08	−0.03∗	−0.08∗∗
	(0.11)	(0.06)	(0.02)	(0.03)
Number of living children	−0.06	−0.10∗∗∗	−0.00	0.02
	(0.05)	(0.03)	(0.01)	(0.02)
Smoking status	0.25∗	−0.38∗∗∗	−0.03	−0.19∗∗∗
	(0.13)	(0.07)	(0.02)	(0.04)
Drinking status	−0.51∗∗∗	0.28∗∗∗	−0.17∗∗∗	−0.35∗∗∗
	(0.12)	(0.07)	(0.02)	(0.03)
Pension insurance participation	−0.55∗	0.72∗∗∗	−0.03	−0.10
	(0.29)	(0.19)	(0.04)	(0.09)
Medical insurance participation	0.09	0.32∗∗∗	0.01	−0.06
	(0.17)	(0.10)	(0.03)	(0.05)
Chronic disease	2.45∗∗∗	0.10	0.74∗∗∗	0.36∗∗∗
	(0.13)	(0.09)	(0.02)	(0.03)
Number of doctors per 1000 persons	0.26∗	−0.04	0.01	0.03
	(0.14)	(0.08)	(0.02)	(0.04)
Number of residential care beds per 1000 persons	0.09	0.03	0.02	0.04
	(0.26)	(0.14)	(0.04)	(0.06)
City fixed effect	Yes	Yes	Yes	Yes
Year Fixed effect	Yes	Yes	Yes	Yes
R-squared	0.16	0.30	0.13	0.11
Observations	4069	4069	4069	4069

Note: ∗ indicates p < 0.10, ∗∗ indicates p < 0.05, ∗∗∗ indicates p < 0.01.

According to the regression results, changes in control variables generally meet theoretical expectations. For example, age negatively affects the mental and physical conditions of older adults living alone; with the increase in age, the physiological functions of older-aged person increase gradually, the cognitive level is lower, and the self-rated health and ability to live independently will have lower scores. Then, education level improves health because of higher efficiency of investment in health, timely recognition of problems, and the adoption of effective self-care measures. Therefore, the general improvement in mental and physical health for the elderly living alone improves. Interacting with children can improve the mental and physical health of older people living alone. More frequent communication with children enables older adults living alone to feel more emotionally supported and valued, which can alleviate loneliness and fulfill the societal expectation of filial piety. Pension insurance secures financial stability and simultaneously supports the mental health of older people by alleviating anxiety and fostering a stronger sense of self-worth. Long-term management stress and future uncertainty associated with chronic diseases can easily act as a trigger for anxiety and depression, while physical functioning may be impaired.

### 4.2 Endogeneity test

We apply an instrumental variables approach to deal with potential bias because of endogeneity issues for more reliable causal interference from model estimation. On the one hand, many of the factors that influence mental and physical health for older people living alone are unobservable; omitted variable bias thus arises. On the other hand, the association between digital inclusion and the health of older adults living alone may be influenced by reverse causality, where those with better health are more likely to engage in digital technologies. In response to this, this paper chooses “the logarithmic form of household annual communication expenditure” (referred to as communication expenditure) as an instrumental variable (Munyegera & Matsumoto, 2016) for the degree of digital inclusion among older individuals living alone. Regarding digital inclusion, older individuals who live alone must pay a set charge in order to use digital services at home. Broadly speaking, the cost of communication increases with increasing levels of digital inclusion and duration. Moreover, there is no clear evidence establishing the relationship between communication expenses and the physical or mental health of elderly people living alone, which aligns with the exogeneity criteria. The two-stage least squares regression outcomes are displayed in Table 3. The first-stage results indicate that the instrumental variable is both significantly and positively related to the level of digital inclusion among elderly individuals living alone, thus verifying that the instrumental variables meet the correlation criterion. The p-values for the Anderson canon. Corr. LM test are all less than 0.1, indicating the rejection of the null hypothesis that the instrumental variables suffer from under-identification. In addition, the Cragg-Donald Wald F statistics exceed the Stock-Yogo critical value of 16.38, dismissing the weak instrument hypothesis and confirming the appropriate selection of instrumental variables. Accordingly, one has reason to believe that the instrumental variable is valid. It follows from the regression using instrumental variable that, with the estimation of the baseline regression results above, digital inclusion significantly enhances mental and physical health among older adults living alone.

**Table 3:** Estimation results using the instrumental variable method.

Variables	(1)	(2)	(3)	(4)
	Mental health		Physical health	
	CES-D	MMSE	SRH	IADL
Digital inclusion	−1.07∗∗∗	0.47∗∗∗	−0.13∗∗∗	−0.15∗∗∗
	(0.18)	(0.09)	(0.03)	(0.04)
Control variables	Yes	Yes	Yes	Yes
City Fixed effect	Yes	Yes	Yes	Yes
Year Fixed effect	Yes	Yes	Yes	Yes
Observations	4069	4069	4069	4069

**Results of first stage regression**				

Communication expenditure	0.18∗∗∗	0.22∗∗∗	0.18∗∗∗	0.16∗∗∗
	(0.01)	(0.01)	(0.01)	(0.01)
Cragg-Donald Wald F	269.21	258.15	258.06	276.87
Anderson canon. corr. LM statistic P	0.00	0.00	0.00	0.00
Centered R-squared	0.07	0.28	0.08	0.74

Note: ∗ indicates p < 0.10, ∗∗ indicates p < 0.05, ∗∗∗ indicates p < 0.01. In line with Stock and Yogo (2005), an F-statistic greater than 16.38 suggests that the instrumental variables are not weak, as it surpasses the critical value for a 10% bias level.

### 4.3 Robustness checks

#### 4.3.1 Propensity Score Matching

Empirical research on the digital inclusion of older individuals living alone may fail to be fully in line with the presuppositions of random sampling but is more of a “self-selection” result. For instance, choices on digital inclusion are made based on unobservable factors like individual attributes, economic status, and health. This leads to “selection bias”. Such a bias might lead to research findings that would not reflectively stipulate the true effects of digital inclusion on the health of older adults living alone. To address selection bias, the study utilized Propensity Score Matching (PSM) to create a counterfactual framework. First, based on the theoretical basis of PSM (Austin, 2011), the elderly living alone were categorized into two distinct groups based on their level of digital inclusion: those who are digitally included and those who are not. Those digitally integrated were considered the experimental group, and those not digitally integrated the control group; thus, matching individuals in the “digitally integrated” group matched with others in the “non-digitally integrated” group but with similar propensity scores in order to minimize selection bias and maintain a balance in the two groups as regards baseline characteristics. Our goal is to adjust the baseline characteristics of both groups to reduce the influence of selection bias on the findings. Second, the matching procedure involves three common methods: nearest neighbor matching, radius matching, and kernel matching, and subsequently, the average treatment effect is determined. Table 4 reveals that the average treatment effects from all three matching approaches are statistically significant in both mental and physical health contexts, further validating the research findings.

**Table 4:** Robustness test-propensity value matching results.

Matching Methods	CES-D		MMSE		SRH		IADL	
	ATT	T-value	ATT	T-value	ATT	T-value	ATT	T-value
Nearest neighbor	−0.45∗∗∗	−3.08	0.76∗∗∗	9.49	−0.13∗∗	−2.32	−0.13∗∗∗	−5.78
Radius	−0.47∗∗∗	−3.69	0.81∗∗∗	11.64	−0.04∗∗	−2.21	−0.12∗∗∗	−6.75
Nuclear	−0.49∗∗∗	−3.85	0.82∗∗∗	11.74	−0.04∗∗	−2.19	−0.12∗∗∗	−6.75
Control variables	Yes	Yes	Yes	Yes	Yes	Yes	Yes	Yes
Observations	4069	4069	4069	4069	4069	4069	4069	4069

Note: ∗ indicates p < 0.10, ∗∗ indicates p < 0.05, ∗∗∗ indicates p < 0.01; Nearest neighbor element matching is done at 1:1; Radius is set to 0.05 in radius matching.

#### 4.3.2 Excluding samples of epidemic years

The COVID-2019 pandemic swept across the globe in a short time and had far-reaching influences on the economies of different countries in an all-round way. It surely has brought influences on the health of older individuals living alone to a certain extent. As a particularly vulnerable group, older adults who live alone have gone through many challenges in the epidemic, like reduced support from society, increased mental stress, and deteriorated health. Therefore, with a view to minimizing the potential interference that might have been brought about on the findings of the study by the epidemic, this study chose to exclude the data sample for 2020, so as to better evaluate the real impact of digital inclusion on the health of older adults living alone. Excluding data from 2020, as shown in Table 5, the regression coefficients for digital inclusion are statistically significant (p < 0.01) in the sample where the epidemic's confounding factors have been excluded, affirming that digital inclusion positively impacts the mental and physical health of older adults living alone.

**Table 5:** Regression results after sample deletion.

Variables	(1)	(2)	(3)	(4)
	CES-D	MMSE	SRH	IADL
Digital inclusion	−0.18∗∗∗	0.09∗∗∗	−0.03∗∗∗	−0.04∗∗∗
	(0.04)	(0.02)	(0.01)	(0.01)
Control variables	Yes	Yes	Yes	Yes
City fixed effect	Yes	Yes	Yes	Yes
Year Fixed effect	Yes	Yes	Yes	Yes
R-squared	0.16	0.31	0.13	0.12
Observations	2199	2199	2199	2199

Note: ∗ indicates p < 0.10, ∗∗ indicates p < 0.05, ∗∗∗ indicates p < 0.01.

#### 4.3.3 Changing estimation model

To ensure the robustness of the findings, a panel logit model is being employed for supplementary analysis on the effects of digital inclusion on the health of older individuals living alone. Specifically, the following settings were made in this paper: the CES-D scale score of more than 10 was used as the criterion for determining the depression status and assigned a value of 1; the MMSE scale score divided into two groups of high and low according to the sample mean, and converted into a dichotomous variable; for the SRH scale, assigning a value of 1 to the individuals whose health status is lower than average; and for the IADL scale, assigning the value of 1 to the individuals who lived alone if there were difficulties in daily life activities. Table 6 shows the estimation results of the health of older adults living alone, obtained through numerical integration using a panel logit model with a modified econometric approach. Control for covariates stringently in analysis; the results suggest that the positive effect of digital inclusion on the health of elderly people living alone is highly consistent with the benchmark regression outcomes, which can furthermore prove that the benchmark regression result in this paper is robust under different model settings and variable treatments.

**Table 6:** Regression results of the changed estimation model.

Variables	(1)	(2)	(3)	(4)
	CES-D	MMSE	SRH	IADL
Digital inclusion	−0.06∗∗∗	0.10∗∗∗	−0.06∗∗∗	−0.40∗∗∗
	(0.01)	(0.02)	(0.01)	(0.06)
Control variables	Yes	Yes	Yes	Yes
χ^2^	820.49	344.18	672.95	261.86
p	0.00	0.00	0.00	0.00
Observations	4069	4069	4069	4069

Note: Robust standard errors are in parentheses. ∗ indicates p < 0.10, ∗∗ indicates p < 0.05, ∗∗∗ indicates p < 0.01.

### 4.4 Heterogeneity analyses

#### 4.4.1 Educational levels

Those who completed high school or achieved a higher level of education were categorized as the high education group, whereas individuals with education below high school were grouped as the low education group. Table 7 presents the analysis of the varying impacts of digital inclusion on the health of older individuals living alone, based on different education levels. In terms of the depression level, the digital inclusion improvement effect was a little higher for the high education group than that for the low education group by about 0.02 (0.01∗2.28 = 0.02, p < 0.01). This is possibly because a highly educated elderly people living alone has the ability to use the benefits of digital technologies more in increasing his or her social networks and searching for emotional support. In China, online platforms like WeChat and TikTok are crucial for facilitating interaction and engagement among older adults. The highly educated group could use the digital platform more effectively to connect with counseling services and interest dating (Hong et al., 2016). By contrast, the low education group had limited access to relevant emotional support due to slightly lower technological literacy. While on cognitive function improvement, digital inclusion tends to affect the low education group slightly higher compared to the high education group by about 0.05 (0.02∗2.28 = 0.05, p < 0.01), which could be that highly educated older individuals have a higher baseline of cognitive abilities, resulting in a smaller marginal gain from learning new skills; the low education group has more room for improvement in cognition after being exposed to content such as online learning and puzzle games through digital inclusion. For example, the “Help for the Elderly by Wisdom” initiated by the China Association of the Elderly in 2021 has helped many low educated elderly learn new knowledge; at the same time, the process of learning has contributed to the development of cognitive ability to some extent. In the case of IADL, digital inclusion had a significant positive effect (α = −0.04, p < 0.01) on the high education group but not on the low education group. This may reflect the privilege of highly educated older adults in using digital technology to optimize their life management and improve their self-care ability, which will enhance their convenience and independence considerably (Czaja & Lee, 2007). A lack of digital literacy and low technological adaptability among less-educated older adults restrict its usefulness in real life.

**Table 7:** Results of heterogeneity in educational levels.

Variables	High education group				Low education group			
	CES-D	MMSE	SRH	IADL	CES-D	MMSE	SRH	IADL
Digital inclusion	−0.18∗∗∗	0.12∗∗∗	−0.02∗∗∗	−0.04∗∗∗	−0.17∗∗∗	0.14∗∗∗	−0.02∗∗∗	−0.01
	(0.02)	(0.01)	(0.00)	(0.00)	(0.02)	(0.01)	(0.00)	(0.00)
Control variables	Yes	Yes	Yes	Yes	Yes	Yes	Yes	Yes
City fixed effect	Yes	Yes	Yes	Yes	Yes	Yes	Yes	Yes
Year fixed effect	Yes	Yes	Yes	Yes	Yes	Yes	Yes	Yes
R-squared	0.13	0.18	0.15	0.09	0.14	0.24	0.15	0.11
Observations	305	305	305	305	3764	3764	3764	3764

Note: Robust standard errors are in parentheses. ∗ indicates p < 0.10, ∗∗ indicates p < 0.05, ∗∗∗ indicates p < 0.01.

#### 4.4.2 Income levels

Based on the average household per capita household income, households with higher income are classified as the high income group, while those with lower income are classified as the low income group. Table 8 provides an analysis of the variation in the effect of digital inclusion on the health of seniors living alone across income groups. This, in terms of improvement effect on the depression level and cognitive functioning, the improvement effect of digital inclusion is about 0.05 (0.02∗2.28 = 0.05, p < 0.01) higher in the high income group than in the low income group. Such might emanate from the sufficiency of economic resource availability. For example, the rates of device ownership in such households were higher among high income older adults living alone; it was easier for high income older adults living alone to afford subscriptions to online mental health support services, like counseling, meditation apps, or cognitive training games, thereby benefiting more in the medium to long term. In this way, highly digitalized tools can place the high income older adults living alone in the middle of a wide circle of information resources, social connections, and health supports to alleviate loneliness and improve mental health. As for SRH, the improvement effect in the low income group is 0.02 (0.01∗2.28 = 0.02, p < 0.01) higher than that in the high income group. On the one hand, it might be because the low income older adults living alone are more sensitive to health management, and health information and services provided through digital technology can prominently fill up the health service gap for them and have greater practical effects. On the other hand, low income older adults who live alone have higher psychological expectations for health improvement. This has brought them a huge psychological fulfillment in the use of digital technology, which in turn enhanced subjective health evaluation. By comparison, the health status of the high income group was relatively better; therefore, health improvement was relatively limited. Even digital technology brought them some improvement, the magnitude of improvement appeared to be small due to their high baseline levels. In terms of IADL, digital inclusion has a significant facilitating effect (α = −0.02, p < 0.01) on the high income group but not on the low income group. A possible explanation for this is that elderly individuals from high income households are more likely to receive training or technical assistance in using digital technologies, enabling them to apply these tools effectively in their daily activities, such as shopping, managing finances, and enhancing their independence and quality of life. In contrast, with limited resources, the low income group may face not only the device-purchasing barrier but also the usage-skill barrier. Besides, different from the high income group, who can extend the digital technologies to many aspects to improve the quality of life, the low income group probably cares more about basic necessities.

**Table 8:** Results of income heterogeneity analysis.

Variables	High income group				Low income group			
	CES-D	MMSE	SRH	IADL	CES-D	MMSE	SRH	IADL
Digital inclusion	−0.16∗∗∗	0.12∗∗∗	−0.01∗∗∗	−0.02∗∗∗	−0.14∗∗∗	0.10∗∗∗	−0.02∗∗∗	−0.01
	(0.02)	(0.01)	(0.00)	(0.00)	(0.03)	(0.02)	(0.00)	(0.01)
Control variables	Yes	Yes	Yes	Yes	Yes	Yes	Yes	Yes
City fixed effect	Yes	Yes	Yes	Yes	Yes	Yes	Yes	Yes
Year fixed effect	Yes	Yes	Yes	Yes	Yes	Yes	Yes	Yes
R-squared	0.15	0.30	0.16	0.11	0.14	0.26	0.10	0.09
Observations	3077	3077	3077	3077	992	992	992	992

Note: ∗ indicates p < 0.10, ∗∗ indicates p < 0.05, ∗∗∗ indicates p < 0.01.

### 4.5 Mechanisms

#### 4.5.1 Life satisfaction

Digital technology has an impact on certain fields, including consumption choices, leisure activities and the values of the population (Lyons et al., 2018). In light of this, column (1) of Table 9 presents an evaluation of the connection between digital inclusion and life satisfaction. The results indicate that digital inclusion can improve health by increasing life satisfaction for older individuals living alone.

**Table 9:** Results of the analysis of mechanisms.

Variables	(1)	(2)	(3)
	Life satisfaction	Utilization of preventive health care services	Social participation
Digital inclusion	0.01∗∗∗	0.01∗∗∗	0.03∗∗∗
	(0.00)	(0.00)	(0.00)
Control variables	Yes	Yes	Yes
City fixed effect	Yes	Yes	Yes
Year fixed effect	Yes	Yes	Yes
R-squared	0.05	0.10	0.08
Observations	4069	4069	4069

Note: Robust standard errors are in parentheses. ∗ indicates p < 0.10, ∗∗ indicates p < 0.05, ∗∗∗ indicates p < 0.01.

The following are three possible reasons. Firstly, digital technologies have made it possible for older persons who are living alone to access timely policy interpretation and information related to their daily life through. This reduces the anxiety caused by information asymmetry, helps them better adapt to modern life, and improves life satisfaction. Secondly, digital inclusion has greatly enriched the leisure and entertainment choices of the elderly. For example, through short-video platforms, apart from passive viewing, elderly people living alone can also enjoy videos of their own interest and even to show themselves and develop their content. These ways of participatory recreation in their daily life have added much psychological satisfaction. Finally, digital technology can also provide channels for older people living alone to express their personal views, which can help them to improve their self-efficacy (Shaw & Gant, 2002), and in turn increase their life satisfaction. It has been established in earlier research that life satisfaction is integral to mental health, as it limits stress and alleviates negative emotions, thereby fostering better mental health (Keyes et al., 2002). Steptoe et al. (2015) found that individuals who have higher life satisfaction have healthier physiological markers, such as lower levels of inflammation and better cardiovascular function. In sum, digital inclusion can affect the health of older adults living alone through pathways of life satisfaction, thereby supporting hypothesis 2a.

#### 4.5.2 Utilization of preventive health care services

The health belief model states that some of the most important elements influencing older people's decision to use health screening services are their expectations regarding the advantages of medical services and their understanding of health hazards (Rosenstock, 1974). Column (2) of Table 9 also shows that digital inclusion increases the use of preventive health services by older people living alone. The likely reason for this is that digital technologies are to some extent able to break down information barriers and facilitate resource sharing across time and geographical constraints. On the one hand, digital technology lowers the threshold of information access, such as online health information and medical apps that provide more convenient access to health information, which not only makes them realize the importance of preventive healthcare, but also motivates them to pay active attention to medical checkups and health monitoring. On the other hand, family members or healthcare professionals can collect the health data of older persons living alone through smart devices, so that they can better understand the physical condition and health risks of older persons, pay attention to their health at all times, and intervene in a timely manner for possible health problems, thus promoting the participation of older persons living alone in preventive healthcare services. It is also confirmed by studies that health checkups help identify potential health problems (Kahana et al., 2002). At the same time, the use of preventive healthcare services not only promotes better health outcomes for seniors but also alleviates some of the financial strain caused by rising healthcare costs, thereby improving their overall quality of life (Kim et al., 2014). In short, digital inclusion can increase access to health services for older individuals living alone, thereby improving the health of the same group, thereby supporting hypothesis 2b.

#### 4.5.3 Social participation

With the increasing availability of internet and new media, the lives of older adults have been greatly enhanced, allowing them to have broader social networks, which in turn promotes social adaptability and improves the level of social participation. Column (3) of Table 9 further confirms that digital inclusion can improve the health of older people living alone through life satisfaction. The reasons for this are that, on the one hand, digital communication channels, represented by social media and instant messaging tools, provide older persons living alone with convenient ways to get in touch with each other, helping them to overcome physical barriers and enabling them to keep in touch with their friends more effectively and to build a wider circle of social relationships. On the other hand, digital technology has expanded the forms of social participation. Through online communities and virtual volunteering, older persons living alone can break through the constraints of time and space and participate in more diversified social activities, such as taking part in online community activities, volunteering or joining interest groups. This not only enriches their daily lives, but also further enhances their sense of belonging to and participation in society. Further research has documented that active social participation reduces the incidence of depression, generalized anxiety disorder, and cognitive impairment, and decreases the risk of physical disability (Golden et al., 2009). Moreover, social engagement can enhance the capability of the elderly to undertake daily activities and ease functional limitations due to physical discomfort (Richard et al., 2013). In essence, digital inclusion contributes greatly to enhancing the health of older adults living alone, through stimulating their social participation, thereby supporting hypothesis 2c.

## 5 Discussion

Using data from CHARLS in 2015, 2018, and 2020, this paper has presented an analysis of the influence of digital inclusion on the health of older adults living alone. Our analysis investigates the multifaceted impact of digital inclusion, offering one of the rare empirical insights into its effects on older adults living alone. It not only confirms the potential advantages of digital inclusion in enhancing the mental and physical well-being of older adults living alone but also uncovers the possible mechanisms through which it operates. Despite the existence of studies indicating possible harmful effects of internet use for older adults (Nowland et al., 2018; Szabo et al., 2019), the findings of this study provide robust evidence that the use of digital technology enhances the health of older adults living alone through three key pathways: improving life satisfaction, encouraging the use of preventive health care services, and facilitating social participation.

Several epidemiological studies have consistently shown that non-co-residing living arrangements are strongly linked to higher levels of depressive symptoms among older adults (Lee & Hong, 2016; Lin & Wang, 2011). Therefore, this paper remains distinguishable from the conventional way of researching middle-aged and older adults in their entirety by targeting those older adults living alone and further elucidating how digital inclusion influences mental and physical health in this age group. Specifically, for mental health, digital inclusion reduced depression levels and improved the cognitive function of the elderly living alone significantly. More specifically, with every one standard deviation increase in the digital inclusion, the depression score decreased by 0.48 (α = −0.21, p < 0.01), which is 4.7% of the mean depression score, whereas the MMSE score increased by 0.27 (α = 0.12, p < 0.01), which is 2.5% of the average MMSE score. The results corroborate those found in earlier studies (Hill et al., 2015; Sen et al., 2022), which indicated that digital technology can positively influence mental health by enabling older adults living alone to communicate with the outside world. Another study in the Chinese context further confirmed that internet use had significant positive effects on older adults’ mental health, including a significant reduction in depression (β = −0.275) and improved cognition (β = 0.300) (Chai et al., 2024). The effects of digital technology appear to be smaller among older adults living alone compared to the overall older adult population. The probable reason for this is that the higher loneliness accompanying living alone neutralizes some of the positive effects of digital inclusion. This has also been demonstrated in other studies. For example, the European Longitudinal Study of Older Adults (ELSA) revealed that although increased internet usage reduced social isolation among the elderly, simply having access to the internet did not entirely eliminate loneliness (Stockwell et al., 2021). From a theoretical standpoint, findings from this study are upheld by two important hypotheses. First, the stress hypothesis posits that during leisure and social activities, emotional needs are satisfied while internet is a medium for enhancing brain stimulation by promoting social interactions (Fratiglioni et al., 2004). The second theory is that of cognitive reserve hypothesis, which projects that the cognitive involvement provided by internet lowers the risk of brain decline, and older persons improve their cognitive ability by learning and researching online (Stern, 2002).

Accordingly, The study went ahead to show the significant effect of digital inclusion on the SRH and IADL scores of older adults who are alone. The analysis showed that for every 1 increase in the digital inclusion score, there was a 0.02 decrease in the SRH score, and for every 1 standard deviation increase in the digital inclusion, there was a 0.11 decrease in the IADL score, which is 1.8% of the IADL average. This observation supports the conclusions of earlier studies (Wen et al., 2023). Older adults living alone are at a higher risk of feeling lonely due to the lack of family support for co-residence, and this may be heightened during times of social isolation, such as the COVID-19 epidemic (Sepúlveda-Loyola et al., 2020). Loneliness not only aggravates health problems but may also influence the extent to which the healthcare needs of older adults are met. In this respect, digital technologies are a valuable way to compensate for the shortage of social support in elderly living alone (Riadi et al., 2022). Studies have found that increased digital literacy and device use significantly improves the physical health of older people living alone, which may be due to their ability to access health information and support services more easily (Levy et al., 2015). Additionally, telemedicine has shown significant effectiveness in improving the health of older adults living alone, particularly during pandemics, where it serves as a vital tool for addressing health issues and accessing healthcare services (Doraiswamy et al., 2020). Not only does telemedicine provide older individuals with a safe channel to access healthcare, but it also strengthens their ability to manage health through virtual consultations and health tracking. Together, these results demonstrate that digital inclusion improves the health of elderly individuals living alone, with significant implications for handling public health emergencies.

The effect of digital inclusion in the health of older people living alone differs by income and education level groups. It is indicated that, among the high income elderly living alone, digital technology is effective in improving IADL, while there was a limited effect of improving IADL for both low income and low education groups. This may be associated with the fact that these groups with low income and education generally have a low dependence on various resources and, therefore, can make relatively few additional benefits resulting from the use of digital technologies (Van Dijk & Hacker, 2003). This group is more accustomed to managing health with traditional resources, and therefore digital technologies are not improving the quality of life for them as much as they do for other groups. At the same time, older persons living alone with lower levels of education may have improved access to information and support because of digital technologies, leading to a significant improvement in their cognitive performance. This finding is supported to some extent by previous studies (Chan et al., 2016; Small et al., 2009). Older people living alone with low levels of education tend to access health information and social support through traditional means, and digital technology provides them with easy access to not only healthcare resources, but also richer learning and cognitive stimulation through online platforms. Particularly in terms of CES-D and MMSE, the benefits of digital inclusion for this group may be more prominent. In addition, the low income group showed slightly higher self-assessed health improvements than the high income group, suggesting that digital technology provides easier and more effective access to health information for older adults with limited financial resources.

This paper is not only to investigate the overall impact of digital inclusion on mental and physical health in older adults living alone but also to explore its possible mechanisms. Second, multi-period panel data is used for analyses, which can better control time-invariant individual heterogeneity than cross-sectional data. Moreover, the empirical study in this paper, which is based on the context of China, contributes to the existing literature on digital technology adoption and the health of older people around the world, especially the practical support for digital inclusion interventions for the group of older people who are living alone, and will provide an important reference for future policy design in developing countries.

Despite several valuable findings from this study, there are some limitations. This paper, first of all, relies on observational data. While it has controlled for some potential confounders, it has been able to under-explore the relationship between digital exclusion and health inequalities among older people, unable to completely rule out causality reversal due to the limitations of the data itself. For example, excessive use of internet can affect the health condition of older people, particularly in cases where loneliness and lack of social interaction force older people living alone to increase their internet use; the latter relationship deserves further exploration. Future studies could adopt more rigorous experimental designs or data with longer time spans to better reveal the causal mechanism of this relationship. Second, research into facilitators and inhibitors of digital inclusion among older adults, in particular, older adults living alone, is insufficient. While current studies have focused on the contribution of digital technology itself, in real life economic condition, digital literacy, and other multiple factors combine to result in health among older adults. Hence, ongoing research is vital to understand how these various factors shape the outcomes of digital inclusion, with a particular emphasis on its effects on the mental and physical health of elderly individuals living alone.

Finally, this research sample in the paper is limited to the Chinese context, and although the localized perspective provides a more accurate practical guide for the study, there may be some difference in the effects and needs of digital inclusion among different cultural contexts' older people's groups. Therefore, the findings of this study can be further validated in the future in other cultural contexts, especially in other developing countries, for exploration of the impacts of different socio-economic conditions, cultural practices, and policy environments on the effects of digital inclusion.

## 6 Conclusions

In general, this study has three main findings. First, digital inclusion can significantly improve the mental and physical health of the elderly living alone. This will, in turn, mean that as an important tool for accessing information and social interaction, digital technologies have the potential to ameliorate the health problems in the elderly living alone. Second, the role of digital inclusion varies across income groups or levels of education. Improvements in IADL were greater for those with higher income and educational attainment. Lastly, mechanistic analyses indicated that digital inclusion could exert positive influences on the health of older adults living alone by enhancing life satisfaction, increasing the use of preventive health care, and facilitating social participation. In response to these results, The following recommendations should be considered. First, the promotion by the government and the social organizations of digital literacy training to help them be surmounted by technological barriers. Second, the improvement of community network infrastructure was greatly emphasized, especially in far-reaching areas, so more elderly living alone could use and gain health benefits from this new technology. Third, promote telemedicine and online psychological counseling services in concert with offline health interventions, paying special attention to the health needs of the elderly living alone.

## CRediT authorship contribution statement

**Yong Yan:** Writing – review & editing, Writing – original draft, Resources, Project administration, Methodology, Funding acquisition, Data curation, Conceptualization. **Huixia Xing:** Writing – review & editing, Visualization, Supervision, Software, Methodology, Conceptualization.

## Informed consent

Our study did not include patient experiments, there is no need for patient consent.

## Ethics statement

This study used data from the China Health and Retirement Longitudinal Survey (CHARLS), which was approved by the Biomedical Ethics Committee of Peking University (IRB00001052-11015).

## Funding

This work has been funded by the Joint Graduate Program of 10.13039/501100004543China Scholarship Council (Project No. 202406640072).

## Declaration of competing interest

The authors declare that they have no known competing financial interests or personal relationships that could have appeared to influence the work reported in this paper.

## Data Availability

This research utilized publicly accessible data from the China Health and Retirement Longitudinal Study (CHARLS) dataset, available at: https://charls.pku.edu.cn/en/.
